# Impact of Gene Polymorphism rs2275913 and Serum IL-17A Levels on Liver Fibrosis Severity Across the Natural History of Chronic Hepatitis B in Indonesia

**DOI:** 10.3390/diseases14070227

**Published:** 2026-06-25

**Authors:** Ummi Maimunah, Andrio Palayukan, Juniastuti, Brahmana Askandar Tjokroprawiro, Muhammad Miftahussurur

**Affiliations:** 1 Doctoral Program of Medical Science, Faculty of Medicine, Universitas Airlangga, Surabaya 60286, Indonesia; ummi.maimunah@fk.unair.ac.id; 2 Division of Gastroenterohepatology, Department of Internal Medicine, Faculty of Medicine, Dr. Soetomo Teaching Hospital, Universitas Airlangga, Surabaya 60131, Indonesia; 3 Department of Internal Medicine, Faculty of Medicine, Dr. Soetomo Teaching Hospital, Universitas Airlangga, Surabaya 60131, Indonesia; 4 Department of Medical Microbiology, Faculty of Medicine, Universitas Airlangga, Surabaya 60131, Indonesia; 5 Department of Obstetrics and Gynecology, Faculty of Medicine, Dr. Soetomo Teaching Hospital, Universitas Airlangga, Surabaya 60131, Indonesia; 6 Helicobacter Pylori and Microbiota Study Group, Institute of Tropical Disease, Universitas Airlangga, Surabaya 60115, Indonesia

**Keywords:** chronic hepatitis B, liver fibrosis, IL-17A, Th17, SNP

## Abstract

Background: A complex interplay between viral activity and host immune responses drives the progression of liver fibrosis in chronic hepatitis B. The T helper 17 (Th17) immune pathway, which produces the pro-inflammatory cytokine interleukin-17A (IL-17A), has been implicated in hepatic fibrogenesis. However, the relationship between IL-17A levels, IL-17A G197A (rs2275913) gene SNP, and the degree of liver fibrosis across different phases of the natural history of chronic hepatitis B remains insufficiently explored. Methods: This study employed an analytical observational design with a cross-sectional approach in treatment-naïve patients with chronic hepatitis B. The degree of liver fibrosis was assessed using liver elastography. IL-17A (rs2275913) gene SNP was analysed using Real-Time PCR, while serum IL-17A levels were measured using enzyme-linked immunosorbent assay. Statistical analyses included Spearman’s correlation, the contingency coefficient, the Chi-square test, the Kruskal–Wallis test, and the Mann–Whitney test, with a significance level set at *p* < 0.05. Results: A total of 76 patients with chronic hepatitis B were included in this study. The phase of disease progression was significantly associated with the degree of liver fibrosis (*p* = 0.016). Median IL-17A levels increased in parallel with fibrosis severity (*p* = 0.003), with a particularly significant association observed during the R phase (*p* = 0.002). However, no significant association was found between the IL-17A G197A (rs2275913) gene SNP and either liver fibrosis severity or serum IL-17A levels. Conclusions: Elevated serum IL-17A levels were associated with greater liver fibrosis severity, particularly during the reactivation phase of chronic hepatitis B. These findings suggest a potential relationship between IL-17A-mediated immune responses and liver fibrosis in patients with chronic hepatitis B.

## 1. Introduction

Chronic hepatitis B virus infection remains a major global health burden, particularly in developing countries. In 2022, hepatitis B was responsible for approximately 1.1 million deaths worldwide, predominantly due to liver cirrhosis and hepatocellular carcinoma. The World Health Organization estimates that around 254 million people were living with chronic hepatitis B in the same year, with approximately 1.2 million new infections occurring annually [1,2]. Chronic hepatitis B infection induces persistent hepatic inflammation, which drives progressive fibrogenesis, ultimately leading to excessive extracellular matrix deposition and architectural distortion of the liver, culminating in cirrhosis [3]. This condition not only increases morbidity and mortality but also significantly impairs patients’ quality of life due to complications associated with advanced liver disease [4]. Globally, cirrhosis ranks as the 11th leading cause of death, while liver cancer is the 16th, together accounting for approximately 3.5% of all deaths worldwide [5,6]. Therefore, early detection and identification of key risk factors associated with fibrosis progression in patients with chronic hepatitis B are essential to prevent the development of advanced liver disease [7].

According to the European Association for the Study of the Liver (EASL) Clinical Practice Guidelines, the natural history of chronic hepatitis B is classified into five phases based on virological, serological, biochemical, and histological characteristics: HBeAg-positive chronic infection, HBeAg-positive chronic hepatitis, HBeAg-negative chronic infection, HBeAg-negative chronic hepatitis, and the HBsAg-negative phase [8]. These phases reflect the dynamic interaction between viral replication and host immune responses and may evolve over time according to changes in viral activity and host immunological control [8,9]. For simplicity, the present study refers to these phases as immune tolerant (IT), immune clearance (IC), low replicative (LR), and reactivation (R) phases, corresponding to the traditional nomenclature commonly used in clinical practice. Antiviral treatment decisions are not determined solely by disease phase but also consider HBV DNA levels, alanine aminotransferase activity, fibrosis severity, age, and other clinical risk factors, in accordance with international guideline recommendations [8,10].

The prevalence of liver fibrosis among patients with chronic hepatitis B varies across different regions worldwide. Epidemiological studies indicate that approximately 20–30% of patients with chronic hepatitis B develop significant fibrosis progression [11]. In developing regions, particularly in Asia and Africa, the prevalence of liver fibrosis is reported to be higher than in developed countries, reaching up to 30–40% or more [12]. In Indonesia, however, data on the prevalence of liver fibrosis in chronic hepatitis B patients remains limited. The progression of liver fibrosis in chronic hepatitis B is known to be influenced by multiple factors, including both viral determinants and host-related factors [7,9]. Among host factors, genetic variation, particularly single-nucleotide polymorphisms (SNPs) in genes regulating immune responses, has been increasingly recognised as a key contributor to fibrosis severity. One such gene is IL-17A, which plays an important role in modulating inflammatory pathways.

Liver fibrosis in chronic hepatitis B is primarily driven by persistent immune-mediated liver injury rather than direct viral cytopathic effects. Chronic exposure to viral antigens promotes activation of innate and adaptive immune responses, leading to repeated hepatocyte injury and repair. This process stimulates hepatic stellate cells, which produce excessive extracellular matrix proteins and collagen, ultimately resulting in progressive fibrosis and architectural distortion of the liver [4,9,13]. Pro-inflammatory cytokines, including IL-17A, IL-6, TNF-α, and transforming growth factor-β (TGF-β), play important roles in regulating these fibrogenic pathways through the activation of hepatic stellate cells and the promotion of extracellular matrix deposition [12,13,14,15]. Among these mediators, IL-17A has attracted increasing attention because it links adaptive immune activation to hepatic stellate cell activation and extracellular matrix accumulation, suggesting a potential role in liver fibrosis associated with chronic hepatitis B [12,14,16].

IL-17 is a family of pro-inflammatory cytokines primarily produced by Th17, playing a crucial role in inflammatory processes and host immune responses to infection [17]. Emerging evidence suggests that increased IL-17 expression is associated with greater severity of liver fibrosis in patients with chronic liver diseases [18]. Among the IL-17 family members, IL-17A and IL-17F are the most extensively studied, exerting their biological effects through interaction with the IL-17RA and IL-17RC receptors [16]. In patients with chronic hepatitis B infection, an increased proportion of circulating Th17 cells has been linked to greater hepatic injury. Th17 cells produce a range of pro-inflammatory cytokines, including IL-17, which amplify inflammatory responses and may contribute to the progression of hepatic fibrogenesis [19].

Several studies have suggested that genetic variations in the IL-17A gene may influence cytokine expression. A study conducted in Iran in 2020 reported that IL-17A gene expression was significantly higher in patients with chronic hepatitis B compared to healthy controls (*p* = 0.0013). Similarly, a 2015 study from China in 2015 demonstrated that the IL-17A G197A (rs2275913) SNP was associated with an increased risk of liver cirrhosis. However, the underlying mechanisms linking this polymorphism to IL-17A regulation remain unclear [20]. In addition, IL-17A levels have been shown to increase significantly during the active phase of disease compared to the inactive Phase in chronic hepatitis B infection (*p* = 0.000). These findings suggest that the IL-17A G197A (rs2275913) gene SNP may not only enhance inflammatory activation but also contribute to disease progression and the persistence of hepatitis B virus infection [21].

Previous studies investigating IL-17A polymorphisms and liver fibrosis have reported inconsistent findings across different populations. While several studies suggested that rs2275913 may increase susceptibility to advanced liver disease and cirrhosis, others failed to demonstrate a significant association with fibrosis severity. These discrepancies may reflect differences in ethnicity, sample size, disease stage distribution, and underlying immune responses. Therefore, further studies in underrepresented populations, including Southeast Asian cohorts, are needed to better understand the potential role of IL-17A genetic variation in chronic hepatitis B-related liver fibrosis.

Despite these findings, evidence directly linking the IL-17A gene SNP to the degree of liver fibrosis remains limited. Most previous studies have focused on its association with advanced clinical outcomes, such as cirrhosis or hepatocellular carcinoma, rather than earlier stages of fibrosis progression. Furthermore, data on the impact of IL-17A G197A (rs2275913) gene SNP on liver fibrosis severity in Indonesia are scarce. Therefore, investigating the relationship between IL-17A and liver fibrosis severity in patients with chronic hepatitis B is essential to provide a more comprehensive understanding of the role of host genetic factors in disease progression. Identifying relevant genetic determinants may support earlier detection strategies and enable more targeted therapeutic approaches to prevent the progression of liver disease in this population.

## 2. Materials and Methods

### 2.1. Study Design

This study employed an analytical observational design with a cross-sectional approach to examine the association between the IL-17A G197A (rs2275913) gene SNP and the degree of liver fibrosis in patients with chronic hepatitis B.

### 2.2. Study Population and Sample

The study population consisted of treatment-naïve patients with chronic hepatitis B. Participants were recruited from the Gastrohepatology outpatient clinic at the Department of Internal Medicine, RSUD Dr. Soetomo, throughout 2024. A consecutive sampling technique was applied, including all eligible patients who met the inclusion criteria during the study period. Eligible participants were adults aged ≥18 years. Body mass index (BMI) was classified according to the Asia–Pacific criteria. Patients were excluded if they had co-infection with hepatitis C or HIV, decompensated cirrhosis, pregnancy, chronic kidney disease, fatty liver disease, or acute infection.

Antiviral therapy using nucleos(t)ide analogues, such as Tenofovir or Entecavir, is strongly indicated in chronic hepatitis B patients with high inflammatory activity, particularly in IC phase (HBeAg -positive chronic hepatitis or IC phase), characterised by elevated ALT levels above the upper limit of normal (ULN) and HBV DNA >20,000 IU/mL, as well as in Phase IV (HBeAg-negative chronic hepatitis or R phase), which carries a high risk of progression to cirrhosis and hepatocellular carcinoma [22].

### 2.3. Study Variables

Independent Variables: (1) SNP on the IL-17A G197A (rs2275913) gene and (2) Serum IL-17A levels. Dependent Variable: Degree of liver fibrosis across disease phases (Phases I–IV) in patients with chronic hepatitis B. Covariates: Age, Sex, ALT levels, HBV DNA viral load and HBeAg status.

### 2.4. Sample Collection and Laboratory Procedures

#### 2.4.1. Blood Sample Collection

Peripheral venous blood samples were collected from each participant using standard aseptic techniques. The samples were subsequently processed for DNA extraction and genetic analysis.

#### 2.4.2. DNA Extraction

Genomic DNA was extracted from the buffy coat fraction of peripheral blood using the Genomic DNA Mini Kit (Blood/Cultured Cell) (Cat. No. GB100, Geneaid Biotech Ltd., New Taipei City, Taiwan), following the manufacturer’s instructions. Briefly, buffy coat samples were lysed with RBC Lysis Buffer, then with GB Buffer, and incubated at 60 °C. The DNA was then purified using a GD spin column, including sequential washing steps with W1 Buffer and Wash Buffer to remove contaminants. Finally, genomic DNA was eluted using preheated Elution Buffer and stored at −20 °C until further analysis. The IL-17A G197A (rs2275913) SNP represents a single base substitution in the promoter region of the IL-17A gene at position 197.

#### 2.4.3. Genotyping Analysis

The IL-17A G197A (rs2275913) SNP was analysed using real-time polymerase chain reaction (Real-Time PCR). Genotyping was performed using the TaqMan^®^ Pre-Designed SNP Genotyping Assay (Applied Biosystems, Foster City, CA, USA), with assay ID C__15879983_10 for rs2275913. PCR reactions were carried out using 75 ng of genomic DNA as the template in a total reaction volume according to the manufacturer’s protocol. Amplification and allelic discrimination were performed using a TaqMan-based Real-Time PCR method, following standard cycling conditions. Forward Primary (with M13-tailed): 5′-TGT AAA ACG ACG GCC AGT GCT CAG CTT CTA ACA AGT AAG-3′. Reverse Primer (with M13-tailed): 5′-CAG GAA ACA GCT ATG ACC AAG AGC ATC GCA CGT TAG TG-3′.

Amplification was performed using the CFX96 Touch™ Real-Time PCR system (Bio-Rad, Hercules, CA, USA). The PCR cycling conditions for IL-17A included an initial enzyme activation at 95 °C for 20 s, followed by 40 cycles of denaturation at 95 °C for 15 s and annealing/extension at 60 °C for 1 min. Genotypes were determined based on fluorescence signal curves generated during amplification.

#### 2.4.4. Measurement of IL-17A Levels

Serum IL-17A levels were measured using an enzyme-linked immunosorbent assay (ELISA) with the Human IL-17A High Sensitivity ELISA Kit (Cat. No. BMS2017-2HS), following the manufacturer’s instructions. Absorbance was measured using a microplate reader (Bio-Rad Model 680, Bio-Rad Laboratories Inc., Hercules, CA, USA) at 450 nm. IL-17A concentrations were calculated based on a standard curve generated from serial dilutions of human IL-17A standards.

#### 2.4.5. Assessment of Liver Fibrosis

The severity of liver fibrosis was assessed using transient elastography (FibroScan^®^; Echosens™, Paris, France) as a non-invasive surrogate marker of hepatic fibrosis. Liver stiffness measurements were expressed in kilopascals (kPa) and categorised according to the METAVIR scoring system as follows: F0–F1 (mild fibrosis, ≤7.0 kPa), F2–F3 (significant fibrosis, 7.1–12.4 kPa), and F4 (advanced fibrosis, ≥12.5 kPa), based on previously validated cutoff values [10].

#### 2.4.6. Serological Markers

Serological markers, including hepatitis B e antigen (HBeAg) and anti-HBe, were assessed using chemiluminescent microparticle immunoassays. HBeAg levels were measured using the Alinity system (Abbott, Wiesbaden, Germany), while anti- HBe was analysed using the ADVIA Centaur aHBe2 assay (Siemens Healthineers, Erlangen, Germany).

### 2.5. Statistical Analysis

Statistical analyses were performed using appropriate statistical software. Descriptive statistics were used to summarise the characteristics of the study population, presented as mean ± standard deviation or median with interquartile range for continuous variables, and as frequencies and percentages for categorical variables. The association between the SNP and the degree of liver fibrosis was evaluated using Spearman’s rank correlation, the contingency coefficient, the Chi-square test, or Fisher’s exact test, as appropriate. Additionally, group comparisons were performed using the Kruskal– Wallis test and the Mann–Whitney U test. To adjust for potential confounding factors, ordinal regression analysis was performed with liver fibrosis severity as the dependent variable and serum IL-17A levels, age, sex, ALT level, HBV DNA level, HBeAg status, and disease phase as independent variables. All analyses were conducted using IBM^®^ SPSS Statistics version 26 (IBM SPSS Statistics), and a *p*-value < 0.05 was considered statistically significant.

### 2.6. Ethical Approval

This study was approved by the Medical Research Ethics Committee of Dr. Soetomo General Academic Hospital, Surabaya, Indonesia (ethical approval number: 1157/KEPK/XI/2024; approval date: 12 November 2024).

## 3. Results

A total of 76 patients with chronic hepatitis B (Table 1) were included in this study. The majority were aged 41–59 years (47.4%) and male (59.2%), with most participants of Javanese ethnicity (80.3%). Regarding disease phase, patients were predominantly in the R (42.1%) and LR (31.6%) phases of the natural history of chronic hepatitis B. More than half of the patients had an HBV DNA viral load >2000 IU/mL (57.9%), while serological profiles were predominantly HBeAg-negative and anti–HBe–positive (68.4%). Based on liver elastography, 55.3% of patients exhibited mild fibrosis, whereas 44.7% had significant to advanced fibrosis. The distribution of IL-17A G197A (rs2275913) gene SNP showed that the AA genotype was the most prevalent (40.8%), followed by GG (30.3%) and GA (28.9%). The median serum IL-17A level across all subjects was 3.345 pg/mL (range: 0.38–34.81).

As shown in Table 2, there was a significant correlation between the degree of liver fibrosis and the phases of the natural history of chronic hepatitis B (*p* = 0.016; r = 0.414).

Further analysis (Table 3) demonstrated that advanced fibrosis was predominantly observed in patients in the IC and R phases (*n* = 19), both of which represent stages commonly associated with indications for antiviral therapy.

**Figure 1:**
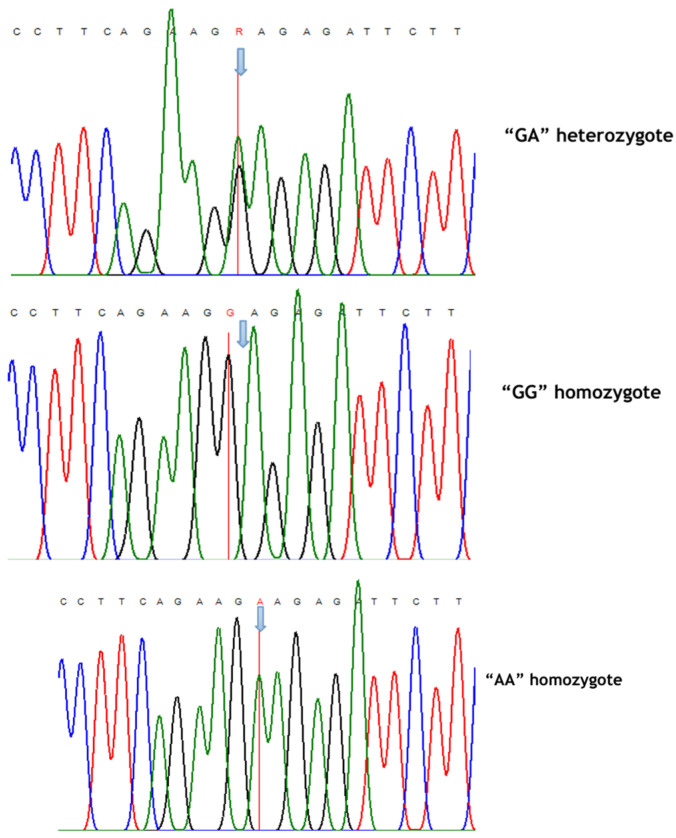
Sequencing of IL-17A G197A (rs2275913) Gene SNP.

As illustrated in Figure 1, SNP sequencing analysis of the IL-17A gene identified three genotype variants based on chromatogram characteristics. The heterozygous GA genotype showed two distinct peaks (black and green), whereas the homozygous variant exhibited single peaks: GG (black) and AA (green).

Further analysis showed no significant association between IL-17A gene SNP and the degree of liver fibrosis (*p* = 0.309; r = 0.244). Although the GG genotype showed a higher proportion of advanced fibrosis (34.8%) than other variants, the relatively uniform distribution across groups suggests that genetic variation at this locus is not a primary determinant of fibrosis severity in this study population (Table 4).

The association between the IL-17A SNP and liver fibrosis severity (Table 5) was further stratified by antiviral treatment indication: non-treatment-indicated phases (IT and LR, *n* = 30) and treatment-indicated phases (IC and R, *n* = 46). In the non-treatment-indicated group, the majority of subjects exhibited mild fibrosis across all genotypes (GG, GA, and AA). In contrast, the treatment-indicated group showed a more pronounced distribution of advanced fibrosis, particularly among individuals with the GG genotype, with 7 of 11 subjects (63.6%) progressing to advanced fibrosis. These findings suggest that during the phases in which antiviral therapy may be indicated in patients within the HBeAg-positive chronic hepatitis and HBeAg-negative chronic hepatitis phases, depending on HBV DNA levels, ALT activity, fibrosis severity, age, and other clinical factors according to international guidelines.

The results demonstrate variability in liver fibrosis severity across the clinical phases of chronic hepatitis B infection, stratified by IL-17A genotype. Table 5, in the IT phase (*n* = 6), all subjects with GG (*n* = 2) and GA (*n* = 2) genotypes exhibited mild fibrosis. In contrast, among those with the AA genotype (*n* = 1), both mild and significant fibrosis were observed. In contrast, during the IC phase (*n* = 14), a marked increase in advanced fibrosis was noted, predominantly in the GG (*n* = 3) and AA (*n* = 3) genotypes.

In the LR phase (*n* = 24), liver conditions appeared relatively stable, with mild fibrosis predominating across genotypes (GG: *n* = 8; GA: *n* = 5; AA: *n* = 6) and a low proportion of advanced fibrosis, consistent with reduced viral replication activity. However, in the R phase (*n* = 32), disease progression became more pronounced, with the GG genotype showing the highest tendency toward advanced fibrosis (*n* = 4), while the AA genotype exhibited the highest prevalence of significant fibrosis (*n* = 5) compared to other genotypes.

To further evaluate the relationship between serum IL-17A levels and liver fibrosis severity in patients with chronic hepatitis B, IL-17A levels were compared across fibrosis categories. The results are presented in Table 6.

As shown in Table 6, median serum IL-17A levels exhibited an increasing trend with greater liver fibrosis severity. The median IL-17A level was 1.995 pg/mL (range: 0.38–28.73) in the mild fibrosis group, 2.575 pg/mL (0.56–34.72) in the significant fibrosis group, and 7.655 pg/mL (1.16–34.81) in the advanced fibrosis group. Correlation analysis demonstrated a statistically significant association between IL-17A levels and fibrosis severity (r = 0.341; *p* = 0.003). To further assess this relationship across different stages of disease progression, IL-17A levels were analysed according to fibrosis severity within each Phase of chronic hepatitis B. The results are presented in Table 7.

The analysis demonstrated that the relationship between serum IL-17A levels and liver fibrosis severity varied across the phases of chronic hepatitis B. Table 7 shows that a statistically significant positive correlation was observed only during the R phase (r = 0.519; *p* = 0.002). In this Phase, IL-17A levels increased markedly with worsening liver damage, with the highest median levels observed in advanced fibrosis (median: 14,380 pg/mL). In contrast, no significant associations were observed in the IT, IC, or LR phases (*p* > 0.05), suggesting that IL-17A levels are more strongly associated with fibrosis severity during the reactivation phase than during other clinical phases of chronic hepatitis B. To evaluate the relationship between IL-17A G197A (rs2275913) gene SNP and serum IL-17A levels, a comparative analysis of IL-17A concentrations across genotype groups was performed. The results are presented in Table 8.

Based on the data presented in Table 8, the analysis showed that the IL-17A G197A (rs2275913) gene SNP (GG, GA, and AA genotypes) was not significantly associated with serum IL-17A levels (*p* = 0.336). Although the GG genotype exhibited the highest median IL-17A level (5990 pg/mL), followed by AA (4130 pg/mL) and GA (2600 pg/mL), post hoc pairwise comparisons (GG vs. GA, GG vs. AA, and GA vs. AA) did not reveal any statistically significant differences (all *p* > 0.05). These findings indicate that genetic variation at the IL-17A G197A (rs2275913) locus does not appear to directly determine serum IL-17A levels in this study population.

To account for potential confounding factors, an ordinal regression analysis was performed including age, sex, ALT level, HBV DNA level, HBeAg status, disease phase, and serum IL-17A level (see Table 9).

**Table 9:** Multivariate Ordinal Regression Analysis of Factors Associated with Liver Fibrosis Severity.

Variable	β	*p*	95% CI
Sex	−0.825	0.132	−1.898 to 0.247
Age	0.026	0.219	−0.015 to 0.067
HBV DNA level	0.146	0.825	−1.146 to 1.438
HBeAg status	−0.363	0.304	−1.054 to 0.329
ALT level	0.006	0.208	−0.003 to 0.015
Serum IL-17A level	0.060	0.031 *	0.006 to 0.114
Disease phase	1.211	0.108	−0.268 to 2.689
Cox & Snell = 0.295			
Nagelkerke = 0.344			
McFadden = 0.179			

* Significant at level 0.05.

After adjustment, serum IL-17A remained independently associated with greater liver fibrosis severity (β = 0.060 (CI 95% 0.006–0.114), *p* = 0.031). In contrast, age (*p* = 0.219), sex (*p* = 0.132), ALT level (=0.208), HBV DNA level (*p* = 0.825), and HBeAg status (*p* = 0.304), disease phase (*p* = 0.108) were not independently associated with fibrosis severity in the multivariable model (all *p* > 0.05). The model explained 34.4% of the variability in fibrosis severity based on the Nagelkerke pseudo-R^2^ value. These findings suggest that elevated serum IL-17A levels are independently associated with more severe liver fibrosis, regardless of demographic, virological, biochemical, and disease-phase characteristics.

## 4. Discussion

The present study demonstrates that the Phase of the natural history of chronic hepatitis B is significantly associated with liver fibrosis severity. In addition, serum IL-17A levels increased in parallel with fibrosis progression, particularly during the R phase. These findings suggest that liver fibrosis severity in chronic hepatitis B is associated not only with viral replication activity but also with host immune responses that may contribute to hepatic inflammation and fibrogenesis.

The natural history of chronic hepatitis B consists of several dynamic phases: IT, IC, LR, and R, which reflect the balance between viral replication and host immune activity. The IC and R phases are typically characterised by heightened immune responses against viral antigens, leading to hepatocellular injury, necroinflammation, and an increased risk of fibrosis progression [8,10]. Therefore, phase-dependent immune activity plays a pivotal role in determining the extent of liver damage and the progression of fibrosis.

From an immunological perspective, one key pathway in this process is the activation of Th17 cells, which produce pro-inflammatory cytokines such as IL-17. This cytokine has been implicated in the pathogenesis of liver fibrosis by activating hepatic stellate cells (HSCs), the principal effector cells in fibrogenesis. IL-17-mediated activation of HSCs promotes the production of extracellular matrix (ECM) components and enhances collagen deposition, thereby contributing to fibrogenic processes [12,13,21].

Moreover, IL-17 amplifies the inflammatory milieu by inducing the release of multiple pro-inflammatory cytokines, including IL-1, IL-6, TNF-α, and IL-13, all of which contribute to HSC activation and liver tissue remodeling [14]. IL-17 has also been shown to recruit myeloid dendritic cells and monocytes, and to stimulate prostaglandin E2 production, further reinforcing the inflammatory and fibrogenic cascade. Collectively, these mechanisms position IL-17 as a critical link between adaptive immune responses and fibrotic processes in the liver.

The finding of increased serum IL-17A levels with greater liver fibrosis severity is consistent with previous studies. Evidence from patients with liver cirrhosis has demonstrated significantly elevated IL-17 levels in both serum and intrahepatic expression, with a positive correlation to fibrosis severity. Notably, IL-17 has been observed to accumulate within fibrotic regions of the liver, suggesting its direct involvement in fibrogenesis [23]. Furthermore, intrahepatic IL-17 expression, as well as serum IL-17 protein levels and IL-17 mRNA expression in peripheral blood mononuclear cells, have been reported to be higher in patients with cirrhosis than in those with chronic hepatitis B or inactive HBsAg carriers [21,22,24]. Genetic studies have also indicated that IL-17 gene SNPs are more frequently detected in cirrhotic patients and are associated with a significantly increased risk of cirrhosis [22]. From a mechanistic perspective, IL-17 has been shown to upregulate the expression of IL-17RA and IL-17RC receptors, while modulating transforming growth factor beta (TGF-β) signalling, a key regulator of hepatic fibrogenesis. Activation of this pathway promotes extracellular matrix production and collagen deposition in the liver [14,21,25]. In addition, IL-17 acts synergistically with other pro-inflammatory cytokines, such as IL-6 and TNF-α, to enhance hepatic stellate cell activation and accelerate fibrosis progression [15,21].

Experimental studies using HepG2 hepatoma cells further support this role, demonstrating that IL-17 can induce fibrogenesis through periostin -mediated collagen deposition, particularly when combined with TNF-α [15]. Moreover, IL-17 contributes to the maintenance of a chronic pro-inflammatory microenvironment by stimulating granulopoiesis through granulocyte-macrophage colony-stimulating factor (GM-CSF). This process may exacerbate hepatocellular injury and promote fibrosis progression in patients with chronic hepatitis [22,23,26].

The findings of this study, which demonstrate the strongest association between IL-17A levels and the degree of fibrosis in the R phase, further support the concept that this Phase is characterised by high hepatic inflammatory activity. During the R phase, viral replication resumes and a stronger immune response to viral antigens develops, which may trigger activation of the Th17 pathway and increase IL-17A production. This condition may be associated with increased inflammatory responses and greater fibrosis severity observed during the reactivation phase. These results are also consistent with a study conducted in Iran, which reported that IL-17A gene expression was significantly higher in patients with chronic hepatitis B compared to the control group (*p* = 0.0013), indicating that activation of the Th17 pathway plays a role in amplifying hepatic inflammatory processes in chronic HBV infection [27].

From an immunogenetic perspective, cytokine-related genetic variation plays a crucial role in modulating inflammatory responses to chronic hepatitis B infection. Polymorphisms in genes encoding IL-17 family cytokines, including IL-17A and IL-17F, have been reported to increase the risk of liver cirrhosis by activating fibrogenic pathways [21]. The novelty of the present study lies in integrating disease phase, IL-17A as an inflammatory biomarker, and fibrosis severity within an Indonesian population, a context that remains underrepresented in the global literature.

In this study, the IL-17A G197A (rs2275913) gene SNP was not significantly associated with either liver fibrosis severity or serum IL-17A levels. The SNP analysis should be considered exploratory because of the relatively limited sample size. This finding suggests that although genetic variation may influence cytokine regulation, its impact on liver disease progression is not necessarily direct. Hepatic fibrogenesis in chronic hepatitis B represents a multifactorial process involving complex interactions between viral activity, host immune responses, and a network of inflammatory mediators.

Accordingly, the effect of a single genetic variant, such as IL-17A G197A (rs2275913), may be insufficient to independently determine fibrosis severity. It is more likely that fibrosis progression is governed by the cumulative and context-dependent effects of multiple genetic and immunological factors, particularly those that are dynamically modulated across different phases of the disease.

An alternative explanation lies in the multifactorial nature of liver fibrosis progression, which involves a broad network of immune mediators, including transforming growth factor beta (TGF-β), IL-6, and TNF-α, as well as complex interactions between viral factors and host genetics [13]. In addition, the activity of the Th17 cells pathway may vary across different disease phases, suggesting that the contribution of IL-17A to fibrogenesis is not uniformly dominant throughout the course of the disease. Previous studies have reported associations between IL-17 gene SNP and susceptibility to hepatitis B virus infection or progression of liver disease. For example, Wang et al. [21] demonstrated that IL-17 gene SNP rs2275913 and rs763780 were associated with increased susceptibility to chronic hepatitis B infection. Similarly, Ge et al. [22] reported that the AA genotype of the IL-17A G197A gene SNP was associated with an increased risk of progression from chronic hepatitis B to liver cirrhosis. However, evidence across studies remains inconsistent. Several investigations have found that IL-17A gene SNP, including rs2275913, as well as IL-17F rs763780, are not consistently associated with chronic hepatitis B or cirrhosis across different populations [28]. These discrepancies may reflect population-specific genetic backgrounds, differences in disease phase distribution, and variations in environmental or immunological contexts.

The IL-17A (rs2275913) gene SNP, characterised by a G-to-A transition in the promoter region (−197G >A), has been implicated in the pathogenesis of chronic hepatitis B infection by influencing IL-17A production, a potent pro-inflammatory cytokine. The G allele, particularly the GG genotype, has been associated with increased susceptibility to HBV infection and a higher risk of progression to liver cirrhosis in patients with chronic hepatitis B [29].

In the present study, the IL-17 gene SNP was not correlated with circulating IL-17A levels, suggesting that variation at the rs2275913 locus is not a sole or primary determinant of systemic IL-17A concentrations in these patients. This finding indicates that other, more dominant multifactorial mechanisms are likely involved in regulating cytokine expression. Serum cytokine levels often reflect the dynamic inflammatory state or clinical Phase of the disease, such as viral load or the extent of liver injury, rather than static genetic profiles alone. In patients with chronic hepatitis B, fluctuations in immune activity across disease phases may obscure the contribution of a single genetic variant. These results further suggest that SNPs within the IL-17A promoter region do not necessarily translate directly into systemic protein levels. It is plausible that the functional effects of rs2275913 are more pronounced at the transcriptional level within local hepatic tissue rather than in circulating serum, or that their impact requires interaction with other cytokines or genetic variants (epistasis) to produce measurable phenotypic differences. These findings provide an important perspective for the global literature, highlighting that in endemic populations such as Indonesia, static genetic variation does not necessarily dictate the dynamic behaviour of circulating pro-inflammatory cytokines in patients with chronic hepatitis [30].

Several limitations should be considered when interpreting these findings. First, the cross-sectional design precludes establishing temporal or causal relationships between IL-17A, genetic variation, and liver fibrosis severity. Second, the relatively small sample size, particularly after stratification by disease phase and genotype, may have limited statistical power to detect modest genetic associations. Therefore, the findings regarding the IL-17A G197A (rs2275913) polymorphism should be interpreted as exploratory. Third, residual confounding from clinical and virological factors cannot be completely excluded. Finally, this was a single-center study conducted in Indonesia, which may limit the generalizability of the findings to other populations.

Future studies should adopt longitudinal designs with larger sample sizes to more comprehensively evaluate the relationship between immune biomarkers, genetic variation, and liver fibrosis progression in patients with chronic hepatitis B. Further research is also warranted to explore the interplay among multiple immune pathways, including key pro-inflammatory cytokines such as transforming growth factor beta (TGF-β), IL-6, and TNF-α, as well as the role of other immune cell populations in hepatic fibrogenesis.

Integrative approaches combining immunological, genetic, and virological perspectives are needed to elucidate the complex mechanisms underlying liver fibrosis progression. In addition, evaluating the potential of IL-17A as a prognostic biomarker or as a target for immunomodulatory therapy represents a promising avenue for future investigation.

The findings of this study provide important insights into the immunological mechanisms underlying liver fibrosis progression in patients with chronic hepatitis B. The observed association between serum IL-17A levels and fibrosis severity suggests that the Th17 immune pathway may contribute to hepatic fibrogenesis. Accordingly, IL-17A may serve as a potential immunological biomarker associated with inflammatory activity and fibrosis severity in patients with chronic hepatitis B. Beyond its diagnostic relevance, the involvement of the Th17 pathway in liver fibrosis pathogenesis also highlights opportunities to develop targeted immunomodulatory therapies targeting specific inflammatory mediators to prevent disease progression.

## 5. Conclusions

This study demonstrated a significant association between clinical disease phase, serum IL-17A levels, and liver fibrosis severity among Indonesian patients with chronic hepatitis B. Higher serum IL-17A levels were observed in patients with more severe fibrosis, particularly during the reactivation phase. However, no significant association was found between the IL-17A G197A (rs2275913) polymorphism and either fibrosis severity or circulating IL-17A levels. These findings suggest that immune-inflammatory activity, reflected by elevated IL-17A levels, may be associated with liver fibrosis severity, whereas this single genetic variant does not appear to independently explain fibrosis severity in this study population. Further longitudinal studies are required to clarify the temporal and causal relationships between IL-17A, immune activation, and fibrosis progression.

## Figures and Tables

**Table 1:** Demographic, Clinical and Laboratory Characteristics of Patients with Chronic Hepatitis B.

Characteristics	*n* (%)	Median (Min-Max)
Age (years old)		
≤40	30 (39.5)	
41–59	36 (47.4)	
≥60	10 (13.2)	
Gender		
Male	45 (59.2)	
Female	31 (40.8)	
Ethnicity		
Javanese	61 (80.3)	
Madurese	9 (11.8)	
Chinese	4 (5.3)	
Buginese	1 (1.3)	
Palembangese	1 (1.3)	
BMI Obesity		
Yes (BMI ≥ 25.00)	6 (7.9)	
No (BMI < 25.00)	70 (92.1)	
Phases		
IT Phase	6 (7.9)	
IC Phase	14 (18.4)	
LR Phase	24 (31.6)	
R Phase	32 (42.1)	
HBV DNA (IU/mL)		
<2000	32 (42.1)	
≥2000	44 (57.9)	
HBeAg(+) Anti-HBe(-)	11 (15.79)	
HBeAg(+) Anti-HBe(+)	9 (11.84)	
HBeAg(-) Anti-HBe(+)	52 (68.42)	
HBeAg(-) Anti-HBe(-)	4 (5.26)	
ALT (U/L)		
≤40	44 (57.9)	
>40	32 (42.1)	
Fibrosis Severity based on Elastography		
Mild	42 (55.3)	
Significant	12 (15.8)	
Advanced	22 (28.9)	
IL-17A Gene SNP		
GG	23 (30.3)	
GA	22 (28.9)	
AA	31 (40.8)	
Serum IL-17A levels		3345 (0.38–34.81)

**Table 2:** Association Between Natural History Phases of Chronic Hepatitis B and Liver Fibrosis Severity.

Phase	*n*	Liver Fibrosis Severity			r	*p*-Value
		Mild	Significant	Advanced		
IT Phase	6	5 (83.3)	1 (16.7)	0 (0.0)	0.414	0.016
IC Phase	14	4 (28.6)	2 (14.3)	8 (57.1)		
LR Phase	24	19 (79.2)	2 (8.3)	3 (12.5)		
R Phase	32	14 (43.8)	7 (21.9)	11 (34.4)		

r = Contingency Coefficient, Chi-Square.

**Table 3:** Association Between Disease Phase (Inactive vs. Active) and Degree of Liver Fibrosis in Chronic Hepatitis B Patients.

Phase	*n*	Liver Fibrosis Severity			r	*p*-Value
		Mild	Significant	Advanced		
Phases without antiviral treatment indication (IT and LR)	30	24 (80.0%)	3 (10.0%)	3 (10.0%)	0.378	0.002
Phases with antiviral treatment indication (IC and R)	46	18 (39.1%)	9 (19.6%)	19 (41.3%)		

r = Contingency Coefficient, Chi-Square.

**Table 4:** Association Between IL-17A Gene SNP and Liver Fibrosis Severity in Chronic Hepatitis B Patients.

IL-17A SNP	*n*	Liver Fibrosis Severity			r	*p*-Value
		Mild	Significant	Advanced		
GG	23	14 (60.9)	1 (4.3)	8 (34.8)	0.244	0.309
GA	22	13 (59.1)	3 (13.6)	6 (27.3)		
AA	31	15 (48.4)	8 (25.8)	8 (25.8)		

r = Contingency Coefficient, Chi-Square.

**Table 5:** Association Between IL-17A Gene SNP and Liver Fibrosis Severity Stratified by Antiviral Treatment Indication.

Phases	*n*	Liver Fibrosis Severity			r	*p*-Value
		Mild	Significant	Advanced		
Phases without antiviral treatment indication (IT and LR) (*n* = 30)						
GG	12	10 (83.3%)	1 (8.3%)	1 (8.3%)	0.257	0.715
GA	8	7 (87.5%)	0 (0%)	1 (12.5%)		
AA	10	7 (70.0%)	2 (20.0%)	1 (10.0%)		
Phases with antiviral treatment indication (IC and R) (*n* = 46)						
GG	11	4 (36.4%)	0 (0%)	7 (63.6%)	0.310	0.300
GA	14	6 (42.9%)	3 (21.4%)	5 (35.7%)		
AA	21	8 (38.1%)	6 (28.6%)	7 (33.7%)		

r = Contingency Coefficient, Chi-Square.

**Table 6:** Association Between Serum IL-17A Levels and Liver Fibrosis Severity.

Liver Fibrosis Severity	*n*	Serum IL-17A Levels	r	*p*-Value
		Median (Range)		
Mild	42	1995 (0.38–28.73)	0.341	0.003
Significant	12	2575 (0.56–34.72)		
Advanced	22	7655 (1.16–34.81)		

r = Spearman Correlation.

**Table 7:** Association Between Serum IL-17A Levels and Liver Fibrosis Severity Across Phases of Chronic Hepatitis B.

Liver Fibrosis Severity	*n*	Serum IL-17A Levels	r	*p*-Value
		Median (Range)		
**IT ** **Phase (** ***n*** ** = 6)**				
Mild	5	2180 (0.77–28.73)	−0.393	0.441
Significant	1	1510 (1.51–1.51)		
Advanced	0	0.0 (0.0–0.0)		
**IC Phase (** ***n*** ** = 14)**				
Mild	4	2495 (0.85–12.89)	0.316	0.271
Significant	2	13.105 (1.07–25.14)		
Advanced	8	4930 (1.73–34.81)		
**LR Phase (** ***n*** ** = 24)**				
Mild	19	1760 (0.63–26.28)	0.188	0.380
Significant	2	8740 (2.51–14.97)		
Advanced	3	5200 (1.16–22.04)		
**R Phase (** ***n*** ** = 32)**				
Mild	14	2380 (0.38–11.39)	0.519	0.002
Significant	7	2640 (0.56–34.72)		
Advanced	11	14,380 (1.24–28.04)		

r = Spearman Correlation.

**Table 8:** Association Between IL-17A Gene SNP and Serum IL-17A Levels.

IL-17A Gene SNP	*n*	Serum IL-17A Levels	*p*-Value	Post Hoc		
		Median (Range)		GG vs. GA	GG vs. AA	GA vs. AA
GG	23	5990 (0.63–28.73)	0.336	0.170	0.282	0.564
GA	22	2600 (0.38–27.18)				
AA	31	4130 (0.56–34.81)				

*p* value = Kruskal–Wallis, Post Hoc = Mann–Whitney.

## Data Availability

All data are available upon request.
